# Mast Cells May Regulate The Anti-Inflammatory Activity of IL-37

**DOI:** 10.3390/ijms20153701

**Published:** 2019-07-29

**Authors:** Theoharis C. Theoharides, Irene Tsilioni, Pio Conti

**Affiliations:** 1 Laboratory of Molecular Immunopharmacology and Drug Discovery, Department of Immunology, Tufts University School of Medicine, Boston, MA 02111, USA; 2 Sackler School of Graduate Biomedical Sciences, Tufts University School of Medicine, Boston, MA 02111, USA; 3 Department of Internal Medicine, Tufts University School of Medicine and Tufts Medical Center, Boston, MA 02111, USA; 4 Immunology Division, Postgraduate Medical School, University of Chieti, 65100 Pescara, Italy

**Keywords:** chemokines, cytokines, IL-37, inflammation, mast cells, neuropeptides

## Abstract

Mast cells are unique immune cells involved in allergic reactions, but also in immunity and inflammation. Interleukin 37 (IL-37) has emerged as an important regulatory cytokine with ability to inhibit immune and inflammatory processes. IL-37 is made primarily by macrophages upon activation of toll-like receptors (TLR) leading to generation of mature IL-37 via the action of caspase 1. In this review, we advance the premise that mast cells could regulate the anti-inflammatory activity of the IL-37 via their secretion of heparin and tryptase. Extracellular IL-37 could either dimerize in the presence of heparin and lose biological activity, or be acted upon by proteases that can generate even more biologically active IL-37 forms. Molecules that could selectively inhibit the secretion of mast cell mediators may, therefore, be used together with IL-37 as novel therapeutic agents.

## 1. Mast Cells in Inflammation

Mast cells derive from bone marrow progenitors and mature perivascularly in all tissues [1], where they are involved in allergic reactions [2]. Mast cells also act as sensors of environmental stress [3].

In addition to allergens, mast cells are also stimulated by pathogens [4], drugs, foods, heavy metals, and “danger signals” [2], as well as certain neuropeptides including corticotropin-releasing hormone (CRH) [5], neurotensin (NT) [6] and substance P (SP) [7,8]. Both NT [9,10] and SP [11,12,13,14] are known to participate in inflammatory processes. Stimulated mast cells can secrete numerous bioactive mediators [15,16,17], utilizing different secretory pathways [18]. Some of these mediators are prestored in secretory granules such as histamine, tryptase and tumor necrosis factor (TNF) [19,20]; others are synthesized de novo and include leukotrienes, prostaglandins, chemokines (CCXL8, CCL2) and cytokines [20,21], that include pro- and anti-inflammatory members, [22]. Many mediators can be secreted from mast cells selectively without degranulation [23]. In particular, CRH stimulates cultured human mast cells to produce vascular endothelial growth factor (VEGF) without tryptase [5].

As a result, mast cells are not only critical for allergic reactions [2,24], but are also important in innate and acquired immunity [25,26], antigen presentation [27,28] and inflammation [29,30].

## 2. IL-37 as An Anti-Inflammatory Agent

The IL-1 family comprises of IL-1a, IL-1b, IL-18, IL-33, IL-36a, IL-36b, IL-36g, IL-37, and IL-38 [31]. Interleukin-37 (IL-37, formerly IL-1F7) belongs to the IL-1 family of cytokines [7,32,33] and is a natural suppressor of immunity and inflammation [21,34,35].

Five isoforms (a–e) have so far been identified [34]. The “b” isoform of IL-37 used here is the most commonly used, but the d isoform was also reported to inhibit the expression of pro-inflammatory cytokines in PBMCs [36]. A specific receptor has not yet been identified for IL-37. A number of studies reported that extracellular IL-37 binds to the alpha chain of the IL-18 receptor (IL-18Rα) [37,38], but with much lower binding affinity than that of IL-18 [39]. 

Both the precursor and mature IL-37 bind IL-18Rα [39]. In addition, IL-37 binds to an IL-18 binding protein (IL-18BP) [40], and to the decoy receptor 8 (IL-R8) [41] via which extracellular forms of IL-37 inhibit innate inflammation in vitro and in vivo [42]. Extracellularly, the IL-37 monomer is the active form involved in reducing innate immunity [43]; instead, homodimerization of IL-37 reduces its anti-inflammatory activity [44]. The precise inhibitory mechanism of action of IL-37 is presently not known. One possibility may be that it inhibits mammalian target of rapamycin (mTOR) [45] since this complex was reported to be involved in the stimulatory action of NT on human microglia [46]. Another possibility may be that IL-37 inhibits inflammasome activation as reported in murine aspergillosis [47]. 

There have been apparently contradicting findings of increased IL-37 in inflammatory states reported in the literature. For instance, IL-37 was reported to be increased in the brain and plasma of patients after ischemic stroke and protected them from inflammatory brain injury [48]. Other studies also showed elevated serum IL-37 concentration in patients with sepsis [49] and in ankylosing spondylitis [50]. Instead, a state of IL-37 deficiency has been reported in calcific aortic stenosis [51]. 

Increased gene expression of IL-37 was associated with suppression of IL-1β and IL-6 production from peripheral blood mononuclear cells (PBMCs) from subjects with systemic inflammatory diseases [22,50,52,53,54,55]. IL-37 has been reported to inhibit the generation of pro-inflammatory cytokines in vitro [56], as well as in vivo [57], but apparently require the IL-1 family decoy receptor IL-1R8 [58]. 

## 3. Mast Cell-Derived Heparin and Tryptase May Regulate IL-37

IL-37 is made primarily by macrophages in response to toll-like receptor (TLR) activation, following which, an IL-37 precursor (pro-IL-37) is cleaved by caspase-1 into mature IL-37. Some of this IL-37 enters the nucleus while the rest is released along with pro-IL-37 outside the cells [59] where both are biologically active. It was recently reported that extracellular IL-37 is active as the monomer, while binding to heparin promotes its homodimerization, with the IL-37 dimers blocking the activity of the IL-37 monomer [43]. Extracellular proteases, hypothesized to be secreted by macrophages, can process pro-IL-37 into a much more biologically active form as shown for recombinant IL-37b with the N-terminus Val46 (V46-218) [60]. 

**Figure 1:**
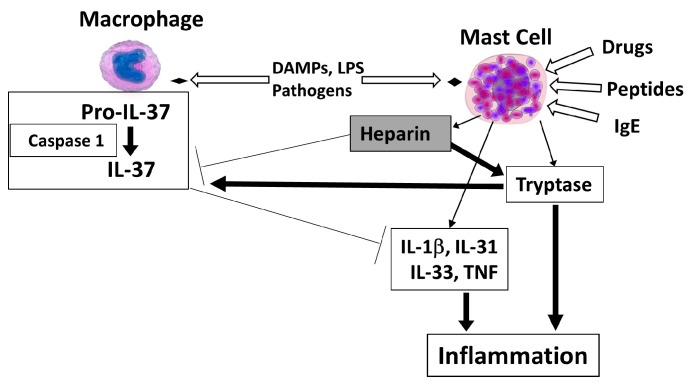
Diagrammatic representation of the role of mast cell-derived heparin in the regulation of the activity of IL-37. Activation of caspase 1 in macrophages, in response to TLR activation, leads to cleavage of pro-IL-37 to mature IL-37, both of which are secreted outside the cell and have anti-inflammatory activity. In the tissue microenvironment, mast cells secrete heparin, which interacts with IL-37 and promotes the formation of inactive homodimers. Mast cells also secrete the proteolytic enzyme tryptase, which exists as homotetramer bound to heparin and promotes inflammation by acting on protease-activated receptors (PAR). In the absence of heparin, biologically active tryptase monomers may be able to generate IL-37 forms with increased anti-inflammatory activity. Open arrows = activation; thin arrows = secretion; thick arrows = stimulation; T arrows = inhibition.

Mast cells are the richest source of heparin [61] and the only source of tryptase [62] in the body. Mast cells could regulate the anti-inflammatory activity of IL-37 in different ways (Figure 1). Heparin will inhibit the action of IL-37 by promoting the creation of homodimers [43]. Moreover, heparin would stabilize the tryptase homotetramer that would promote inflammation via activation of protease-activated receptors (PAR) [63]. Instead, tryptase monomers could generate mature, superactive IL-37 [60], in a method analogous to what had been reported for IL-33 [64,65]. 

## 4. Conclusions

We believe that the ratio of IL-1 to IL-37 is a determining factor in inflammatory diseases. Several drugs targeting IL-1β or its soluble IL-1R are available for treating inflammatory conditions [66], but there is still a need for more effective management of inflammation. IL-37 would be superior to other biologics beacause it is capable of inhibiting the generation of both cytokines and chemokines. IL-37 may also be administered together with other natural molecules [67,68], such as the flavonoid tetramethoxyluteolin, which has been reported to inhibit mast cell release of cytokines [8,69].

## Abbreviations

CRH	corticotropin releasing hormone
CXCL8	chemokine (C-X-C Motif) ligand 8
DAMPs	damage-associated molecular patterns
IL	interleukin
LPS	lipopolysaccharide
NT	neurotensin
PBMCs	peripheral blood-derived mononuclear cells
PAR	protease-activated receptors
SP	substance P
TNF	tumor necrosis factor
